# Advances in Corticosteroid Therapy for Ocular Inflammation: Loteprednol Etabonate

**DOI:** 10.1155/2012/789623

**Published:** 2012-03-28

**Authors:** Timothy L. Comstock, Heleen H. DeCory

**Affiliations:** Global Medical Affairs, Pharmaceuticals, Bausch & Lomb Inc., 1400 North Goodman Street, Rochester, NY 14609, USA

## Abstract

Topical corticosteroids are effective in reducing anterior segment inflammation but are associated with adverse drug reactions (ADRs) including elevation of intraocular pressure (IOP) and cataract formation. Retrometabolic drug design has advanced the development of new corticosteroids with improved therapeutic indices. Engineered from prednisolone, loteprednol etabonate (LE) has a 17**α**-chloromethyl ester, in lieu of a ketone group, and a 17**β**-etabonate group. LE is highly lipophilic and binds with high affinity to the glucocorticoid receptor; any unbound LE is metabolized to inactive metabolites. LE has been studied in several anterior segment inflammatory conditions (giant papillary conjunctivitis, allergic conjunctivitis, anterior uveitis, and keratoconjunctivitis sicca), and in postoperative ocular inflammation and pain. Combined with tobramycin, it is effective in blepharokeratoconjunctivitis. Elevations in IOP are infrequent with LE, and the absence of a C-20 ketone precludes formation of Schiff base intermediates with lens proteins, a common first step implicated in cataract formation with ketone steroids.

## 1. Introduction

The eye is vulnerable to damage from relatively low levels of intraocular inflammation. The blood-aqueous and blood-retinal barriers usually limit penetration of protein and cells from the peripheral circulation, while regulatory molecules and cells in the eye actively suppress immunologic responses [1]. Nevertheless, ocular inflammatory conditions and surgical trauma induce changes in the blood-aqueous and blood-retinal barriers [1–3]. As a result, immune cells and mediators of inflammation enter the eye, resulting in the classical clinical signs and symptoms of ocular inflammation including redness, pain, swelling, and itching [4]. Ocular inflammation, if left untreated, may lead to temporary or permanent loss of vision [5].

Topical corticosteroids are useful for the management of anterior segment inflammation. Corticosteroids elicit numerous potent anti-inflammatory effects [6]. For instance, they suppress cellular infiltration, capillary dilation, the proliferation of fibroblasts, collagen deposition, and eventually scar formation; they stabilise intracellular and extracellular membranes; and they increase the synthesis of lipocortins that block phospholipase A_2_ and inhibit histamine synthesis in the mast cells. Inhibition of phospholipase A_2_ prevents the conversion of phospholipids to arachidonic acid, a critical step in the inflammatory cascade. Corticosteroids also increase the enzyme histaminase and modulate transcription factors present in mast cell nuclei.

Corticosteroids mediate their anti-inflammatory effects primarily through the modulation of the cytosolic glucocorticoid receptor (GR) at the genomic level [7, 8]. After corticosteroids bind to the GR in the cytoplasm, the activated corticosteroid-GR complex migrates to the nucleus, where it upregulates the expression of anti-inflammatory proteins and represses the expression of proinflammatory proteins. However, recent work suggests that the activated corticosteroid-GR complex also elicits nongenomic effects, particularly the inhibition of vasodilation, vascular permeability, and migration of leukocytes [7, 9]. In addition, corticosteroids mediate anti-inflammatory activity through membrane-bound GR-mediated nongenomic effects and through direct nonspecific interactions with cellular membranes [9, 10].

Because the GR is involved in a plethora of signalling pathways—in fact, more than 5000 genes are expressed or suppressed following glucocorticoid exposure [11]—long-term use or high dosages of corticosteroids can result in adverse drug reactions (ADRs) such as increased IOP [12, 13]. Most studies implicate the involvement of trabecular meshwork (TM) cells and myocilin gene expression in the mechanism of corticosteroid-induced IOP increase. Steroids decrease the outflow of aqueous humor by inhibiting the degradation and/or enhancing the deposition of extracellular matrix material within the TM and/or cross-linking of actin fibres between TM cells [14]. Structural changes in the TM, in turn, result in corticosteroid-induced ocular hypertension, which can progress to secondary iatrogenic open-angle glaucoma [15]. Myocilin, initially referred to as TM-inducible glucocorticoid response or *TIGR* gene product, is a 55-kDa protein induced after exposure of TM cells to dexamethasone for 2-3 weeks, which is also closely associated with decreased aqueous humor outflow and steroid-induced IOP increase [16]. Different mutations within the myocilin gene lead to a variety of glaucoma phenotypes in both juvenile and adult-onset primary open-angle glaucoma, providing further evidence for its role in steroid-induced IOP [14].

**Figure 1:**
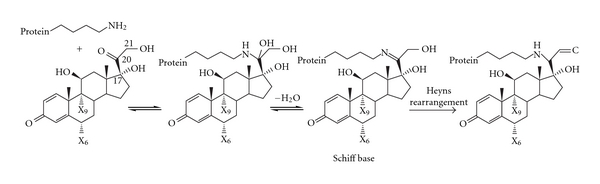
Mechanism of steroid-induced cataract formation adapted from [17].

Another ADR associated with corticosteroid use is the formation of cataract. However, the mechanism of steroid-induced cataract formation appears to be chemically based and not likely to be related to the downstream effects of GR activation. Currently, the most prominent hypothesis for cataract formation involves nonenzymatic formation of Schiff base intermediates between the steroid C-20 ketone group and nucleophilic groups such as *ε*-amino groups of lysine residues of lens proteins [17]. The formation of Schiff bases is followed by a Heyns rearrangement of the adjacent C-21 hydroxyl group, resulting in stable anime-substituted adducts (Figure 1) [17]. While this covalent binding mechanism could account for cataract formation with C-20 ketone-based corticosteroids, other mechanisms of steroid-induced cataract formation may exist. Interestingly, covalent adducts have been observed only in steroid-induced cataract, not in other cataracts.

Further research into the mechanisms of action of steroids—both for their anti-inflammatory effects and for ADRs—is underway. Herein, we review the design of new corticosteroids through retrometabolic design and review available data from preclinical and clinical studies of loteprednol etabonate (LE), the first retrometabolically designed topical steroid to reach marketing status. Studies confirming the premise of retrometabolic design are discussed.

## 2. Retrometabolic Drug Design

Only a small fraction of systemically administered drugs will distribute to the eye from the general circulation, and an even smaller fraction thereof will cross the blood-retinal barrier to reach the eye. Thus, topical administration of corticosteroids is the preferred route for anterior segment inflammatory conditions as it maximizes drug delivery to the anterior segment and minimizes systemic exposure. Topical administration also helps avoid systemic ADRs such as hypothalamic-pituitary-adrenal-(HPA-axis) suppression. Nevertheless, topical ophthalmic corticosteroids are associated with ADRs including elevations in IOP, cataract formation following extended use, delayed wound healing, and lower resistance to infection [1, 7]. As previously discussed, steroid ADRs appear to arise from the continued action of the corticosteroid-GR complex at the genomic level beyond the action required to elicit anti-inflammatory effects or, in the case of cataract formation, through formation of covalent bonds with lens protein.

In an effort to decrease ADRs, Bodor and colleagues developed the concept of retrometabolic drug design more than 30 years ago [18]. The underlying principle of retrometabolic drug design is to synthesize analogues of lead compounds or reference compounds, starting from a known inactive metabolite of that lead compound. The inactive metabolite is converted into an isosteric/isoelectronic analogue with structural modifications designed for rapid, predictable metabolism back to the original inactive metabolite after eliciting the desired therapeutic effect [19] (Figure 3). Although Bodor named such analogues “soft drugs,” these analogues were predicted to have therapeutic potency similar, if not identical, to that of the lead compound, but, due to the structural modifications included by the design, any active drug remaining following attainment of therapeutic effect would be metabolically deactivated, thus minimizing any ADRs (hence, the “soft drug” terminology). However, the increase in therapeutic index could only be achieved if the drug was stable enough to reach its receptor to elicit the desired effect, while any free drug remaining thereafter would be metabolized to avoid ADRs. Metabolism that is too rapid would result in decreased efficacy as would poor bioavailability and/or poor GR-binding affinity. In other words, there had to be a balance between the solubility and lipophilicity of the drug, its tissue distribution and receptor binding, and subsequent rate of metabolic deactivation.

**Figure 2:**
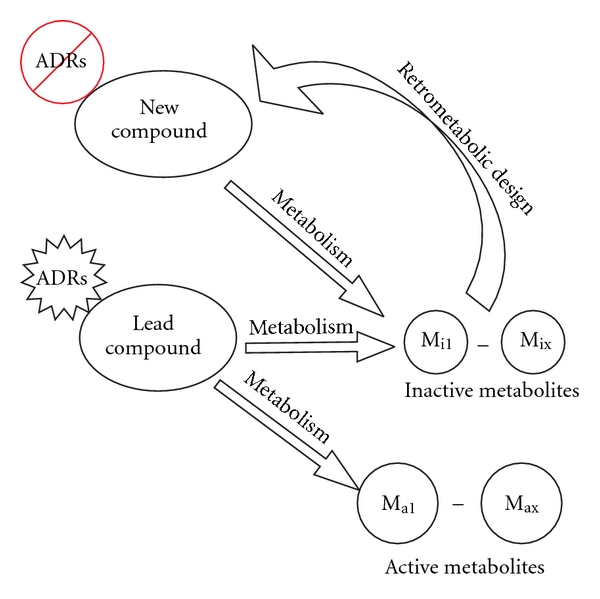
Concept of retrometabolic drug design in which a new lead compound is created based on an inactive metabolite of a previous lead compound.

**Figure 3:**
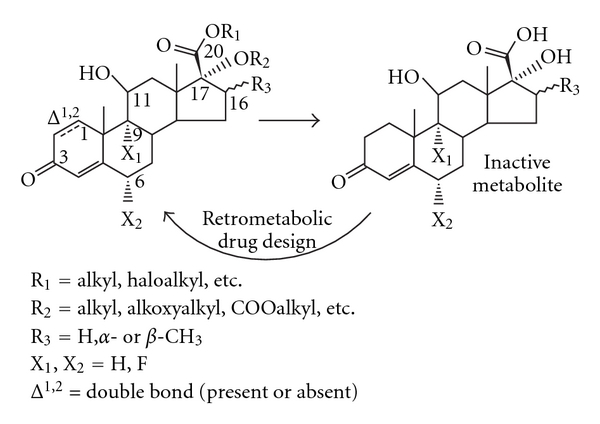
Retrometabolic design of cortienic acid-based derivatives adapted from [52].

Over the years, Bodor and colleagues applied retrometabolic drug design to a variety of therapeutic agents including antimicrobials, *β*-blockers, analgesics, and acetylcholinesterase (ACE) inhibitors, with several retrometabolically designed compounds reaching marketing application. With respect to ocular corticosteroids, Bodor designed a number of analogues, starting with Δ1-cortienic acid, the primary metabolite of prednisolone, that lacks corticosteroid activity [19] (Figure 2). To obtain new lead compounds, the pharmacophore moieties of the 17*α*-hydroxyl and 17*β*-carboxy substituents of the lead compound had to be restored by suitable isosteric/isoelectronic substitution containing esters or other types of functions that restored the original corticosteroid's anti-inflammatory potency while incorporating hydrolytic features to ensure metabolism. Other structural considerations included the presence/absence of double bond at the Δ1 position, fluorination at 6*α* carbon (X_2_) and/or 9*α* carbon (X_1_), and methylation at 16*α* or 16*β* carbons (R_3_). Over a hundred possible drugs were synthesized and tested in preclinical anti-inflammatory models, and structure/activity studies concluded that the best substitutions for maximal activity included a haloester at the 17*β* position and a carbonate or ether at the 17*α* position. 17*α* esters were also considered but were quickly abandoned due to their potential to form mixed anhydrides with the haloesters, and subsequent potential for lens protein binding. Thus, in addition to the C-20 ketone moiety of prednisolone being replaced to avoid the possibility of formation of Schiff base intermediates, other chemical features associated with potential cataractogenesis were also eliminated by design.

## 3. Loteprednol Etabonate

### 3.1. Preclinical Studies

The most promising drug candidate among cortienic acid-based derivatives synthesized by Bodor and colleagues was loteprednol etabonate (LE; chloromethyl 17*α*-ethoxycarbonyloxy-11*β*-hydroxy-3-oxoandrosta-1,4-diene, 17*β*-carboxylate) [20]. LE is the 17*β*-chloromethyl ester of Δ1-cortienic acid with a 17*α*-etabonate moiety and was predicted to undergo rapid deesterification to an inactive carboxylic acid metabolite after exerting its effect, thereby minimizing the likelihood of toxicity.

Selection of LE for further development was based on a number of criteria. LE is highly lipophilic—its lipophilicity is 10 times greater than that of dexamethasone, a characteristic that may increase its efficacy by enhancing penetration through biological membranes [21]. Further, competitive binding studies with rat lung type II GRs demonstrated that the binding affinity of LE was 4.3 times that of dexamethasone [22]. A vasoconstriction test in humans used to assess bioavailability showed that LE produced a blanching response similar to that of betamethasone 17*α*-valerate, thereby confirming good penetration properties and strong potency [12]. But more importantly, initial studies by Bodor showed that the therapeutic index of LE was more than 20-fold better than that of other corticosteroids including hydrocortisone 17*α*-butyrate, betamethasone 17*α*-valerate, and clobetasone 17*α*-proprionate based on the cotton pellet granuloma test and thymolysis potency [9].

**Figure 4:**
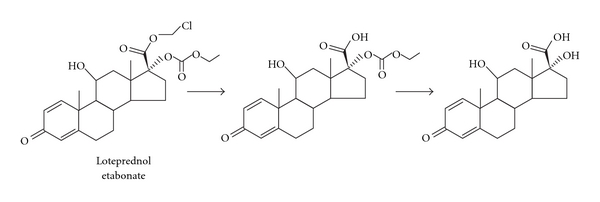
Metabolism of loteprednol etabonate.

Studies in animals confirmed that LE is indeed predictably metabolized by local esterases into its inactive metabolite, Δ1-cortienic acid. Druzgala et al. [23] studied the ocular absorption and distribution of ^14^C-labelled LE 0.5% in the eyes of rabbits. The highest concentrations of LE were found in the cornea, followed by the iris/ciliary body and aqueous humor. The cornea also showed the highest ratio of metabolite to LE, indicating that the cornea was the primary site of metabolism, while aqueous humor concentrations of LE were approximately 100-fold lower. This finding suggested that LE may exert a decreased IOP effect relative to other corticosteroids, as high levels of steroids in the aqueous humor are thought to contribute to decrease outflow through the TM. LE was found to have a terminal half-life (*t*_1/2_) of 2.8 hours in dogs following intravenous administration [24]. Further, when absorbed systemically, LE was found to be metabolized to Δ1-cortienic acid etabonate and then to Δ1-cortienic acid (Figure 4) and eliminated rapidly through the bile and urine [20, 25].

More importantly, a comparison of the IOP-elevating activity of LE with that of dexamethasone in rabbits confirmed a lack of IOP effect with LE [26]. LE and dexamethasone, both at 0.1% concentrations, and vehicle were instilled topically 8 times per day for 2 days to normotensive rabbits in a 3-way crossover design. Treatment with dexamethasone produced an increase in IOP of *∼*4 mm Hg after only 8 instillations, while there was no significant difference in IOP in animals treated with LE versus those treated with vehicle.

More recently, Glogowski and Proksch [27] studied the ocular pharmacokinetics of LE in rabbits with corneal inflammation. Consistent with results obtained by Druzgala et al., high concentrations were found in the cornea and conjunctiva, while low levels were found in the aqueous humor. The *C*_max⁡  _and AUC_(0–24 h)_ were, respectively, 3.62 (5.47) *μ*g/mL and 6.10 *μ*g · h/g in the conjunctiva, 1.40 (1.45) *μ*g/mL and 3.30 *μ*g · h/g in the cornea, and 0.0293 (0.00805) *μ*g/mL and 0.0838 *μ*g · h/g in the aqueous humor. These results confirm good corneal and conjunctival penetration of LE into the anterior segment, while hydrolysis limits significant aqueous humor accumulation. In addition, Samudre et al. studied the efficacy of LE compared to other corticosteroids in a model of ocular inflammation—lipopolysaccharide-induced uveitis in rabbits [28]. It was found that LE 0.5% induced greater GR migration to the nucleus as compared to prednisolone acetate 1% and fluorometholone 0.1%. This effect correlated with the disappearance of inflammatory cells from the corneal stroma and restoration of corneal endothelium.

Numerous additional preclinical studies have been conducted to date on LE in addition to these presented here. Taken together, they demonstrated that LE achieves the required balance between the solubility/lipophilicity, ocular tissue distribution, receptor binding, and subsequent rate of metabolic deactivation outlined by Bodor when he conceptualized retrometabolic drug design.

### 3.2. Clinical Studies: LE Suspension Formulations

Since the design of LE by Bodor and colleagues, 3 ophthalmic suspension formulations of LE have been developed and tested clinically in various ocular inflammatory conditions (Table 1) and postoperative inflammation (Table 2): a 0.2% suspension, a 0.5% suspension, and a combination suspension of LE 0.5% plus tobramycin 0.3%. Clinical safety and efficacy of these formulations is briefly summarized below. These studies confirm the clinical anti-inflammatory potency of LE and lack of significant IOP effects after its use.

Bartlett et al. [29] studied the safety and efficacy of LE 0.5% in the treatment of papillae in contact lens-associated GPC. In this 4-week study, LE-treated patients demonstrated a significant reduction in the primary ocular sign of GPC (papillae, *P* ≤ 0.02) and were rated better in the investigator global assessment (*P* = 0.017) as compared to placebo-treated patients. The mean IOP did not change over the course of the study. The efficacy and safety of LE in the management of GPC associated with contact lens use were further evaluated by Asbell and Howes [30] and Friedlaender and Howes [31] in two identical studies. In both studies, patients received 0.5% LE or placebo 4 times daily for 6 weeks. The proportion of patients with an improvement in papillae severity and itching severity was greater in the LE treatment group than in the placebo treatment group (*P* ≤ 0.001). A significant improvement in contact lens tolerance in the LE treatment group was observed in 1 study (*P* = 0.002). Transient IOP elevations (≥10 mm Hg from baseline) occurred more often in the LE treatment group but were attributed to the reservoir effect of the contact lens, which patients continued to wear for the duration of the study.

Dell et al. studied the efficacy and safety of 0.5% LE administered prophylactically over a period of 6 weeks before the start of the allergy season in patients with SAC [32]. During peak pollen counts, the results of composite severity of itching and bulbar conjunctival injection and the investigator global assessment significantly favoured LE treatment (*P* ≤ 0.001), compared with placebo. An IOP increase of greater than 10 mm Hg was noted in 2 patients receiving placebo and none of the patients treated with LE. The efficacy of LE 0.2% for the treatment of SAC was further evaluated by Dell et al. [33] and Shulman et al. [34] in 2 similar studies. In both studies, LE treatment reduced bulbar conjunctival injection and itching to a greater extent than placebo (*P* ≤ 0.008). No patient experienced elevated IOP of ≥10 mm Hg over baseline in one study, while 1 patient in each treatment group experienced an IOP elevation in the second study. Recently, Elion-Mboussa et al. [35] compared the clinical efficacy and safety of LE 0.2% with that of an antihistamine, olopatadine 0.1%, in patients with acute SAC. It was found that LE 0.2% was superior to olopatadine in reducing both bulbar injection and ocular itching (*P* ≤ 0.0006) following 2 weeks of treatment. No patients experienced a clinically significant increase in IOP (≥10 mm Hg ) over baseline, suggesting that the risk of elevated IOP with LE 0.2% may not differ from that with an antihistamine.

Two clinical studies were conducted to compare the efficacy and safety of LE 0.5% to prednisolone acetate 1.0% in the treatment of anterior acute uveitis [36]. In the first study, study treatments were initially administered 8 times daily and continued QID for up to 6 weeks. While in the second study, study treatments were initially administered 16 times a day and continued QID for up to 4 weeks. Both treatments significantly reduced anterior chamber cell and flare as well as pain and photophobia, compared to baseline. However, a last-observation-carried-forward analysis in the second study showed a greater reduction in cell and flare with prednisolone than with LE (*P* ≤ 0.017), although no differences were found at any on-treatment study visits. Across the 2 studies, only 1 LE-treated patient versus 7 prednisolone-treated patients experienced an IOP increase of >10 mm Hg over baseline (*P* = 0.05) [37].

LE has also been studied in the treatment of dry eye or keratoconjunctivitis sicca. Pflugfelder et al. conducted a pilot study evaluating the efficacy of LE 0.5% versus placebo for the treatment of patients with dry eyes secondary to delayed tear clearance [38]. Although there were significant within-treatment improvements in the primary subjective variable (visual analogue severity for worst symptom at baseline) in both groups, there were no significant within-treatment improvements in the primary objective variable (composite corneal staining) in either treatment group. Further analysis of a subset of patients with moderate-to-severe inflammation showed a significant difference between the LE-treated group and vehicle-treated group in central corneal staining, nasal bulbar conjunctival hyperaemia, and lid margin injection at some visits (*P* < 0.05). None of the patients experienced a clinically significant increase in IOP following 1 month of therapy. LE 0.5% has also been studied as induction therapy for topical cyclosporine ophthalmic emulsion 0.05% in the treatment of patients with dry eye [39]. Cyclosporine improves tear production in patients with ocular inflammation associated with dry eye. However, relief of signs and symptoms is often delayed by 1 to 6 months from the initiation of therapy, and it has been reported that 1 in 5 patients treated with cyclosporine experiences burning and stinging. LE induction therapy administered 2−6 months prior to the institution of long-term cyclosporine treatment decreased stinging and improved compliance when compared with the cohort of patients who were prescribed cyclosporine without LE induction therapy (*P* ≤ 0.04). A follow-up study presented in abstract form indicated that 2 weeks of induction therapy with LE was sufficient to improve subjective and objective parameters, compared to artificial tears alone, thereby accelerating clinical improvement [40].

Two identical placebo-controlled trials examined the safety and efficacy of LE in treating postoperative inflammation following cataract surgery with intraocular lens implantation [6, 41]. Patients were administered 1 drop of LE 0.5% or vehicle in each eye every 4 hours, 4 times daily for up to 14 days. In both studies, greater resolution of anterior chamber inflammation (the sum of anterior chamber cells and flare) was achieved with LE than with placebo (*P* < 0.001). Results for pain resolution, reported separately, [42] indicated that 84% of LE-treated patients, compared to 56% of vehicle-treated patients, across the 2 studies had no pain at the final visit (*P* < 0.05). The mean IOP decreased after surgery in both the LE and placebo treatment groups.

The combination of LE 0.5% and tobramycin 0.3% (LE/T) was evaluated in the treatment of blepharokeratoconjunctivitis (BKC) in 2 studies [43, 44]. Both White et al. and Chen et al. compared the safety and efficacy of LE/T with that of dexamethasone 0.1%/tobramycin 0.3% (DM/T). Subjects in each study were randomized to LE/T or DM/T administered 4 times daily for 14 days. Both steroid combinations were effective in improving the signs and symptoms of BKC relative to baseline (*P* ≤ 0.0001). In both studies, there were no significant differences in the mean change from baseline to day 15 in the signs and symptoms of composite severity, and LE/T was found to be noninferior to DM/T. However, in both studies, DM/T-treated patients experienced a significant increase in the mean IOP when compared with LE/T-treated patients (*P* ≤ 0.0339). IOP increases of ≥10 mm Hg over baseline were reported more often for the DM/T treatment group.

### 3.3. New Formulations of Loteprednol Etabonate

The safety and efficacy of LE ophthalmic ointment 0.5% (LE ointment) in the treatment of inflammation and pain following cataract surgery were studied in 2 randomized, multicentre, double-masked, parallel-group, vehicle-controlled studies [45]. Pooled analysis of the data from these studies showed that significantly more LE ointment-treated patients than vehicle-treated patients had complete resolution of anterior chamber inflammation and no pain at day 8 of treatment (*P* < 0.0001). Fewer LE ointment-treated patients required rescue medication, and fewer had an ocular adverse event.

Studies are also underway on a new gel formulation of LE 0.5% in the treatment of inflammation and pain following cataract surgery (NCT01010633 and NCT01060072). As indicated previously, LE is highly lipophilic with limited solubility in water. A gel formulation could provide improved product homogeneity over a suspension formulation and perhaps a more consistent clinical response as a consequence. Results of these studies are expected to be released in 2012.

## 4. IOP and Cataract Formation with Loteprednol Etabonate

The clinical studies summarized above confirm the efficacy of LE in the treatment of ocular inflammatory disease and postoperative inflammation associated with cataract surgery and are supportive of LE meeting the required balance between the solubility/lipophilicity, ocular tissue distribution, receptor binding, and subsequent rate of metabolic deactivation, all of which are essential features of successful retrometabolic design. Additional studies with LE, including studies in known steroid responders, and additional study analyses further confirm the reduced incidence of ADRs with LE in clinical practice.

Holland et al. [46] compared the steroid-induced IOP effect and other ocular adverse effects of LE/T with those of DM/T in 306 healthy volunteers. In this study, patients were treated 4 times daily for 28 days or longer. The number of patients experiencing IOP increases of ≥10 mm Hg from baseline at any study visit was significantly lower in the LE/T group than in the DM/T group (1.95% versus 7.48%; *P* = 0.028); similar results were observed for mean changes from baseline in IOP (*P* < 0.05 at all visits). Patients in the LE/T group were also more likely to report better ocular comfort/tolerability ratings relative to an artificial tear standard, compared to subjects in the DM/T group [47].

Novack et al. [48] conducted a meta-analysis of the IOP data from LE development studies in which patients were treated with LE, of any concentration, for 28 days or longer. The analysis included a combination of 1648 healthy volunteers and patients with a variety of ocular inflammatory conditions. IOP elevations of ≥10 mm Hg over baseline occurred in 1.7% (15/901) patients using LE, compared to 0.5% (3/583) patients using vehicle and 6.7% (11/164) patients using prednisolone acetate. Excluding subjects that continued to wear soft contact lenses (allowed in the GPC trials and thought to contribute to a reservoir effect), the rates were 0.6%, 1.0%, and 6.7% for LE, vehicle, and prednisolone acetate, respectively. Cheng et al. also conducted a meta-analysis of LE IOP data but included data retrieved from available published LE clinical studies [37]. A total of 1660 patients with a variety of ocular conditions were included in this analysis. In placebo-controlled studies, the IOP elevation rate was 1.7% in the LE group versus 0.6% in the placebo group (*P* = 0.3). In active (prednisolone) comparator studies, the IOP elevation rate was 0.8% in the LE group versus 5.5% in the prednisolone group (*P* = 0.05).

The absence of significant ADRs was further studied by Ilyas et al. who studied the long-term safety of LE 0.2% by conducting a retrospective review of 397 seasonal and perennial conjunctivitis patients who had used LE 0.2% on a daily basis for extended periods of time [49]. Of these patients, 159 had been using LE 0.2% continuously for at least 12 months. There were no reports of posterior subcapsular opacification and no clinically meaningful changes in IOP in this group. In fact, there were no observations of IOP elevations greater than 4 mm Hg over baseline at any time.

**Figure 5:**
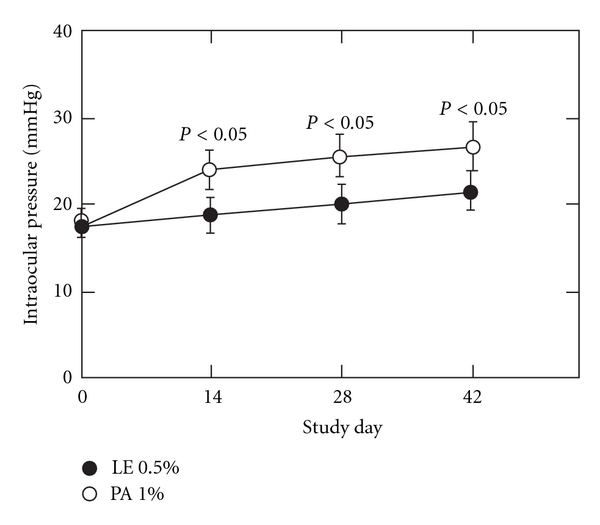
Mean (SEM) IOP for subjects receiving loteprednol etabonate and prednisolone. Within-treatment significant changes from baseline are indicated adapted from [50].

Bartlett et al. [50] compared the effects of LE 0.5% and prednisolone acetate 1.0% on IOP in a crossover study in 19 known steroid responders. Studies in known steroids responders are useful since differences in steroid-induced IOP effects are emphasized in this population. Subjects received either LE 0.5% or prednisolone 1.0% for 42 days followed by a washout period of 14 days prior to being crossed over to the other treatment. During LE treatment, the mean IOPs were within the normal range, with a mean IOP elevation of 4.1 mm Hg over the 42-day period (*P*, not significant). In contrast, during prednisolone treatment, the mean IOP elevation was 9.0 mm Hg (*P* < 0.05, compared to baseline) (Figure 5). Because the study protocol required discontinuation of subjects upon significant IOP elevation, the authors noted that the hypertensive effect of prednisolone may have been underestimated.

Finally, Holland et al. [51] reported the attenuation of ocular hypertension in steroid responders after corneal transplantation. In this retrospective review, 30 post-penetrating keratoplasty and post-keratolimbal allograft patients with IOP increases to ≥21 mm Hg while being treated with prednisolone acetate 1.0% were switched to LE 0.5%. Results showed a mean (SE) reduction of IOP from 31.1 (1.13) mm Hg to 18.2 (1.37) mm Hg (*P* = 0.0001) with no signs of graft rejection after switching treatment from prednisolone acetate to LE.

With respect to cataract formation, as indicated earlier, Manabe et al. showed that C-20 ketone steroids such as prednisolone form covalent bonds with lens protein. These authors further showed that nonketolic analogues were unable to form such adducts. Bodor and colleagues designed LE with a C-20 ester rather than a C-20 ketone, and thus LE is unable to form covalent adducts via this mechanism, although other mechanisms of cataractogenesis cannot be ruled out. Nevertheless, the long-term study by Ilyas et al. did not suggest a potential for cataract formation with LE. Further, a review of global postmarketing adverse event data for LE 0.5% revealed only 7 reports of cataract formation with LE use (data through August 2011, Bausch & Lomb, data on file) since product launch. During that time, an estimated 20 million LE units were distributed globally. Taken together, these data suggest that the rapid metabolism of LE to inactive hydrophilic metabolites in conjunction with the lack of the C-20 ketone have resulted in a steroid with significantly less, if any, potential for promoting cataract formation.

## 5. Conclusions

Retrometabolic drug design principles have led to the development of LE, a C-20 ester corticosteroid. LE appears to achieve the necessary balance between solubility/lipophilicity, tissue distribution, GR receptor binding, and metabolic deactivation to be effective as a topical ophthalmic steroid. LE is safe and effective in treating a wide variety of ocular inflammatory conditions including giant papillary conjunctivitis, seasonal allergic conjunctivitis, and uveitis as well as in the treatment of ocular inflammation and pain following cataract surgery. ADRs such as cataract formation and IOP elevation were minimized with LE owing to its retrometabolic design and their absence confirmed in clinical studies.

## Figures and Tables

**Table 1:** Loteprednol etabonate: summary of randomized, controlled, clinical safety and efficacy studies in ocular Inflammatory diseases.

Ocular disease	Treatment duration and study treatments	Efficacy	Safety	Reference
	4 weeks LE 0.5% (*n* = 55) versus placebo (*n* = 55)	(i) Reduced papillary severity 1–4 (*P* ≤ 0.02 versus placebo) (ii) Investigators global assessment better (*P* = 0.017 versus placebo)	No change in mean IOP in LE treatment group	[29]
				
Giant papillary conjunctivitis	6 weeks LE 0.5% (*n* = 111) versus placebo (*n* = 109)	(i) Reduced papillary severity at final visit (*P* < 0.001 versus placebo) (ii) Reduced itching at final visit (*P* = 0.001 versus placebo) (iii) Improved lens tolerance at final visit (*P* = 0.002 versus placebo)	↑ IOP (≥10 mm Hg): *n* = 3 for LE *n* = 0 for placebo	[30]
				
	6 weeks LE 0.5% (*n* = 109) versus placebo (*n* = 110)	(i) Reduced papillary severity at final visit (*P* = 0.001 versus placebo) (ii) Reduced itching at final visit (*P* < 0.001 versus placebo) (iii) Improved lens tolerance at final visit (*P* = 0.053 versus placebo)	↑ IOP (≥10 mm Hg): 7% versus 0% *n* = 8 for LE *n* = 0 for placebo	[31]

Prophylaxis of seasonal allergic conjunctivitis	6 weeks LE 0.5% (*n* = 145) versus placebo (*n* = 143)	(i) Reduced composite of itching and BCI (*P* = 0.001 versus placebo) (ii) Investigators global assessment better (*P* < 0.001 versus placebo)	↑ IOP (≥10 mm Hg): *n* = 0 for LE *n* = 2 for placebo	[32]

	6 weeks LE 0.2% (*n* = 66) versus placebo (*n* = 67)	(i) Reduced BCI, itching at 2 weeks (*P* ≤ 0.034 versus placebo) (ii) Investigator global assessment at week 2 better (*P* < 0.001 versus placebo)	No ↑ IOP (≥10 mm Hg) ≥1 AE: 68% versus 90% (*P* = 0.002)	[33]
				
Seasonal allergic conjunctivitis	6 weeks LE 0.2% (*n* = 67) versus placebo (*n* = 68)	(i) Reduced BCI, itching at 2 weeks (*P* ≤ 0.008 versus placebo) (ii) Investigator global assessment at week 2 better (*P* < 0.001 versus placebo)	↑ IOP (≥10 mm Hg): *n* = 1 for LE *n* = 1 for placebo No AE: 36% versus 19% (*P* = 0.035)	[34]
				
	2 weeks LE 0.2% (*n* = 151) versus olopatadine (*n* = 149)	(i) Reduced BCI, itching at week 2 in both groups (*P* ≤ 0.0006 in favour of LE)	No ↑ IOP (≥10 mm Hg) ≥1 AE: 2.0% versus 1.3% (*P* = NS)	[35]

Anterior uveitis	6 weeks LE 0.5% (*n* = 36) versus prednisolone 1.0% (*n* = 34)	(i) Resolution of ACC (LOCF): 74% versus 88% (*P* = NS) (ii) Resolution of flare (LOCF): 71% versus 81% (*P* = NS) (iii) Resolution of pain (LOCF): 79% versus 81% (*P* = NS)	↑ IOP (≥10 mm Hg): *n* = 0 for LE *n* = 1 for prednisolone	[36]
			
	4 weeks LE 0.5% (*n* = 84) versus prednisolone 1.0% (*n* = 91)	(i) Resolution of ACC (LOCF): 72% versus 87% (*P* = 0.015 in favour of prednisolone) (ii) Resolution of flare (LOCF): 66% versus 82% (*P* = 0.017 in favour of prednisolone) (iii) Resolution of pain (LOCF): 90% versus 85% (*P* = NS)	↑ IOP (≥10 mm Hg): *n* = 1 for LE *n* = 6 for prednisolone	[36]

Blepharokerato-conjunctivitis	2 weeks LE 0.5%/tobramycin 0.3% (*n* = 136) versus dexamethasone 0.1%/tobramycin 0.3% (*n* = 137)	(i) Improvement from baseline in composite signs and symptoms severity at day 15 in both groups (ii) LE/T noninferior to DM/T in reduced composite signs and symptoms at day 15 (−15.2 [7.3] versus −15.6 [7.7], *P* = NS) (iii) Investigator global assessment: 43.6% versus 40.9% cured (*P* = NS)	↑ IOP (≥10 mm Hg): *n* = 0 for LE/T *n* = 1 for DM/T Mean IOP increase at day 15: −0.1 mm Hg versus 1.0 mm Hg (*P* = 0.0091) ≥1 AE: 2.9% versus 6.5% (*P* = NS)	[43]
			
	2 weeks LE 0.5%/tobramycin 0.03% (*n* = 178) versus dexamethasone 0.1%/tobramycin 0.3% (*n* = 176)	(i) Improvement from baseline in composite signs and symptoms severity at day 15 in both groups (*P* < 0.0001 versus baseline) (ii) LE/T noninferior to DM/T in reduced composite signs and symptoms at day 15 (−11.6 [4.6] versus −12.4 [4.7], *P* = NS)	↑ IOP (≥10 mm Hg): *n* = 6 for LE/T *n* = 13 for DM/T Mean IOP increase at day 15: 1.33 mm Hg versus 2.43 mm Hg (*P* = 0.0039) ≥1 AE: 13.0% versus 23.2%	[44]

Keratoconjunctivitis sicca	4 weeks 0.5% LE (*n* = 32) versus placebo (*n* = 34)	(i) Reduced hyperaemia at week 2 and week 4 (*P* ≤ 0.0473 versus placebo) (ii) Subset analysis in patients with moderate-to-severe inflammation at baseline (iii) Reduced central corneal staining, nasal bulbar conjunctival hyperaemia, and lid margin injection at some visits (*P* < 0.05 versus placebo)	No ↑ IOP (≥10 mm Hg) No significant change in mean IOP ≥1 AE: 16.7% versus 23.5%	[38]

LE: loteprednol etabonate, IOP: intraocular pressure, ACC: anterior chamber cells, AE: adverse event, BCI: bulbar conjunctival injection, LOCF: last observation carried forward, NS: not significant.

**Table 2:** Loteprednol etabonate: summary of randomized, controlled, clinical safety and efficacy studies in postoperative inflammation.

Treatment duration and study treatments	Efficacy	Safety	Reference
2 weeks LE 0.5% (*n* = 109) versus placebo (*n* = 113)	(i) Resolution of ACI at final visit: 64% versus 29% (*P* < 0.001 versus placebo) (ii) Treatment failure rate: 6% versus 30% (*P* < 0.001 versus placebo) (iii) Investigator global assessment of treatment effect (*P* < 0.001 versus placebo) (iv) Grade 0 (no pain) at final visit: 85% versus 54% (*P* = 0.003)	↑ IOP (≥10 mm Hg) *n* = 3 for LE *n* = 0 for placebo Mean IOP decreased in both groups ≥1 AE: 58% versus 80% (*P* < 0.001)	[41, 42]

2 weeks LE 0.5% (*n* = 102) versus placebo (*n* = 101)	(i) Resolution of ACI at final visit: 55% versus 28% (*P* < 0.001) (ii) Treatment failure rate: 7% versus 32% (*P* < 0.001 versus placebo) (iii) Investigator global assessment of treatment effect (*P* < 0.001 versus placebo) (iv) Grade 0 (no pain) at final visit: 83% versus 59% (*P* = 0.018)	↑ IOP ≥10 mm Hg) *n* = 0 for LE *n* = 1 for placebo Mean IOP decreased in both groups ≥1 AE: 54% versus 75% (*P* = 0.002)	[6, 42]

2 weeks LE 0.5% ointment (*n* = 404) versus vehicle (*n* = 401) [2 studies]	(i) Resolution of ACI at day 8: 27.7% versus 12.5% (*P* < 0.0001) (ii) Grade 0 (no pain) at day 8: 75.5% versus 43.1% (*P* < 0.0001) (iii) Need for rescue medication: 27.7% versus 63.8% (*P* < 0.0001)	↑ IOP (≥10 mm Hg): *n* = 3 for LE *n* = 1 for vehicle Mean IOP decreased in both groups Mean IOP decreased in both groups ≥1 AE: 47.2% versus 78.0% (*P* < 0.0001)	[45]

LE: loteprednol etabonate, IOP: intraocular pressure, ACI: anterior chamber inflammation, AE: adverse event.
